# How to measure the entropy of a mesoscopic system via thermoelectric transport

**DOI:** 10.1038/s41467-019-13630-3

**Published:** 2019-12-20

**Authors:** Yaakov Kleeorin, Holger Thierschmann, Hartmut Buhmann, Antoine Georges, Laurens W. Molenkamp, Yigal Meir

**Affiliations:** 1 Department of Physics, Ben-Gurion University of the Negev, Beer Sheva, 84105 Israel; 2 Center for the Physics of Evolving Systems, Biochemistry and Molecular Biology, University of Chicago, Chicago, IL 60637 USA; 3 Kavli Institute of Nanoscience, Faculty of Applied Sciences, Delft University of Technology, Lorentzweg 1, 2628 CJ Delft, The Netherlands; 4 Physikalisches Institut (EP III), Universität Würzburg, D-97074 Würzburg, Germany; 5 Centre de Physique Theorique, Ecole Polytechnique, CNRS, Universite Paris-Saclay, 91128 Palaiseau, France; 6 College de France, 11 place Marcelin Berthelot, 75005 Paris, France; 7 Center for Computational Quantum Physics, Flatiron Institute, 162 Fifth Avenue, New York, NY 10010 USA; 8 DQMP, Universite de Geneve, 24 quai Ernest Ansermet, CH-1211 Geneve, Switzerland; 9 The Ilse Katz Institute for Nanoscale Science and Technology, Ben-Gurion University of the Negev, Beer Sheva, 84105 Israel

**Keywords:** Electronic properties and materials, Quantum Hall, Quantum dots, Quantum information

## Abstract

Entropy is a fundamental thermodynamic quantity indicative of the accessible degrees of freedom in a system. While it has been suggested that the entropy of a mesoscopic system can yield nontrivial information on emergence of exotic states, its measurement in such small electron-number system is a daunting task. Here we propose a method to extract the entropy of a Coulomb-blockaded mesoscopic system from transport measurements. We prove analytically and demonstrate numerically the applicability of the method to such a mesoscopic system of arbitrary spectrum and degeneracies. We then apply our procedure to measurements of thermoelectric response of a single quantum dot, and demonstrate how it can be used to deduce the entropy change across Coulomb-blockade valleys, resolving, along the way, a long-standing puzzle of the experimentally observed finite thermoelectric response at the apparent particle-hole symmetric point.

## Abstract (web summary)

Advances in nanotechnology make it possible to probe traditionally macroscopic notions of thermodynamics at the mesoscopic scale. Here the authors propose a method that can determine the entropy of a quantum dot system from transport measurements.

## Introduction

The entropy of a mesoscopic system can yield non-trivial information on emergence of exotic states, such as two-channel Kondo impurity^1^, non-abelian anyons in the $\nu =5/2$ regime^2,3^, or Majorana modes in topological superconductors^4^. Nevertheless, the measurement of entropy in such small electron-number systems is highly non-trivial. Previous studies^5,6^ used the asymmetry of the in and out tunneling processes in a quantum dot (QD) to determine the degeneracy of the QD states, while recent elegant experiments^7^ have employed the thermodynamic Maxwell relation between entropy evolution and chemical potential, ${(\partial \mu /\partial T)}_{n}=-{(\partial S/\partial n)}_{T}$, in order to directly measure entropy transitions in semiconductor QDs. This latter experiment required measurements of another thermodynamic quantity—the charge of the system as a function of gate voltage, for different temperatures, and hence a specially designed device. Here we propose a different approach to this problem: can one extract information about the entropy from transport measurements? Obviously, this requires a measurement of both particle and thermal (entropy/heat) transport. This question has been addressed in the context of bulk solids^8–11^, with sometimes debated points of view. A general relation exists between the low-temperature thermopower and specific-heat (entropy) of a free electron gas, and this relation appears to apply in a number of materials^9,10^. However, thermopower is, quite generally, a transport coefficient and its relation to entropy has been shown to be questionable in systems with strongly anisotropic transport for instance^11^. In the opposite high-temperature limit, where temperature is the largest energy scale in the system, general relations between the thermopower and derivatives of the entropy can be derived, embodied in the Heikes^8,12,13^ and Kelvin^11,14^ formulas.

Consider an arbitrary mesoscopic system in the Coulomb-blockade regime (where only $N$ and $N+1$-particle states are energetically relevant), whose entropy one wishes to measure. The method we propose here is based on a general observation, which is also an important result of our work: if one weakly couples this system to leads, the conductance of such an interacting system can be put in the form of a non-interacting conductance formula, provided one takes into account a temperature-dependent shift of the chemical potential (gate voltage). The thermal response (TR), in turn, can be written in a similar manner, where the temperature-dependent shift in the chemical potential produces an extra contribution. We show that this extra term, which can be determined by comparing the actual thermal response of the system to that of the related non-interacting system (which can be estimated using a newly introduced high-temperature version of the original Mott formula^15^), can be used to extract the entropy of such a mesoscopic system even in the case of arbitrary spectrum and degeneracies, and then demonstrate the usefulness of the approach by applying it to several model systems. One big advantage of our formulation is that one can apply it to any such mesosopic system where measurements of both electrical conductance and thermopower are available. This allows us to apply our procedure to existing data of thermoelectric response of a single QD, and demonstrate how it can be used to deduce the entropy change and the QD’s degeneracy. In the process we explain the long standing puzzle of the observation of a non-zero thermopower at the apparent electron-hole symmetry point in the Coulomb Blockade (CB) valley^16,17^.

## Results

### General formulation

Consider a general mesoscopic system with many-body eigenstates ${\Psi }_{i}^{(N)}$, where $N$ is the number of electrons in that state, with energies ${E}_{i}^{(N)}$ (with ${g}_{i}^{(N)}$ the degeneracy of the energy ${E}_{i}^{(N)}$), whose entropy one wishes to measure. In order to perturb the system as little as possible, we weakly couple the mesoscopic system to two reservoirs (with coupling ${V}_{i}$ for each state $i$). In this weak-coupling limit ${\Gamma }_{ij}=2\pi {V}_{i}{V}_{j}\rho$, the characteristic level broadening, with $\rho$ the density of states in the reservoirs, obeys ${\Gamma }_{ij}\ll T$, where $T$ is the temperature. In this limit the conductance $G$ through the mesoscopic system can be written as the sum of individual transitions from state $i$ with $N$ electrons to state $j$ with $N+1$ electrons^18^

1 $$G(\mu ,T)=	 \sum _{ij}{G}_{ij}(\mu ,T)=\sum _{ij}{{\mathcal{T}}}_{ij}^{(0)}\times \left[\left({P}_{i}^{(N+1)}(\mu ,T)+{P}_{j}^{(N)}(\mu ,T)\right.\right]\\ 	 \times \frac{df({E}_{i}^{(N+1)}-{E}_{j}^{(N)}-\mu ,T)}{d\mu }$$

where ${{\mathcal{T}}}_{ij}^{(0)}$ is equal to ${\Gamma }_{ij}$ times the overlap of the $N+1$-particle many-body wavefunction ${\Psi }_{j}^{(N+1)}$ with the $N$-particle wavefunction ${\Psi }_{i}^{(N)}$, with the addition of the electron tunneling in from the leads (or the reverse process) (see Supplementary Information, Eq. (1)). In the above $f(E,T)$ is the equilibrium Fermi function, $\mu$ the chemical potential, and ${P}_{i}^{(N)}(\mu ,T)={e}^{-({E}_{i}^{(N)}-\mu N)/T}/Z$ is the equilibrium probability of the system to be in the $N$-particle many-body state $i$, with $Z$ the partition function (except for the experimental part, we use ${k}_{B}=1$ throughout the paper, where ${k}_{B}$ is the Boltzman coefficient, so that temperature has units of energy and entropy is dimensionless). A similar expression can be written for the TR, defined as $dI/dT$, the change in the linear-response current due to temperature difference between the leads, in analogy to conductance, with $df\!/d\mu$ being replaced by $df\!/dT$. We assume that the Coulomb energy is significantly larger than $T$ and $\Gamma$ so that for a given chemical potential, $G$ involves transitions between states with only $N$ or $N+1$ particles. A crucial step in our formulation is the demonstration that the above general expressions for the conductance and the thermal response for an arbitrary interacting system can be accurately written, in the vicinity of each $N\to N+1$ transition, as those for a non-interacting system, but with a temperature-dependent effective chemical potential (see Supplementary Note 1):

2 $${G}_{ij}(\mu ,T)=C(T){G}_{ij}^{NI}(\mu +{\Delta }_{ij}(T),T)$$

where ${G}_{ij}^{NI}$ is the conductance for a non-interacting system with same spectrum and couplings, and $C(T)$ is some temperature-dependent prefactor, that will drop out when the relation between G and TR is derived. This temperature-dependent shift in the chemical potential is given by

3 $${\Delta }_{ij}(T)=\frac{{E}_{j}^{(N+1)}-{E}_{i}^{(N)}}{2}+\frac{T}{2}{\mathrm{log}}\left[\frac{\sum _{j}{g}_{j}^{(N+1)}{e}^{-{E}_{j}^{(N+1)}/T}}{\sum _{i}{g}_{i}^{(N)}{e}^{-{E}_{i}^{(N)}/T}}\right]$$

In the simple case of a transition from an empty state into a single level, with degeneracy $g$, this shift reduces to $\frac{1}{2}T\,{\mathrm{log}}\, g$, which has been noticed before^3,19^, and has been measured experimentally^5^. In that case this shift was attributed to the fact the chemical potential has to shift in order to compensate for the fact there are $g$ ways for an electron to tunnel into the QD, while having a single channel for tunneling out, an asymmetry that has been verified experimentally^5,20^. In contrast, our expression indicates that in the case of many levels, which has not been discussed before, the temperature-dependent part of the shift does not depend on which level the electron tunnels through, and what its degeneracy is. This part of the shift is identical for all transitions, and is equal one half of the difference of the canonical free energies between the CB valleys corresponding to $N$ and $N+1$ electrons.

The explicit dependence of ${\Delta }_{ij}$ on $T$ allows us to write, in a similar manner to Eq. (2), an explicit expression for the TR of a general interacting system in terms of its conductance and the TR of the related non-interacting system,

4 $${{\rm{TR}}}_{ij}(\mu ,T)=C(T){{\rm{TR}}}_{ij}^{NI}(\mu +{\Delta }_{ij}(T),T)+{G}_{ij}(\mu ,T){\Delta }_{ij}(T)/T$$

In order to derive an equation for ${{\rm{TR}}}^{NI}$, the thermal response of a non-interacting system with same spectrum and couplings, we generalize the Mott formula^15^, valid for $T\ll \Gamma$, to the regime $T\gg \Gamma$ (see Eq. (5) in the Methods section and Supplementary Note 2 for derivation). Thus, the deviation of the ${\rm{TR}}$ from ${{\rm{TR}}}^{NI}$ (calculated from the conductance) allows us to estimate ${\Delta }_{i,j}(T)$, and consequently the entropy difference between the consecutive CB valleys: $\Delta {S}_{N\to N+1}=2d{\Delta }_{ij}(T)/dT$.

So, given the experimentally or numerically obtained $G(\mu ,T)$ and $TR(\mu ,T)$, the procedure we propose for finding the entropy difference between consecutive CB valleys is the following: (1) Given $G(\mu ,T)$, one can use our variant of the Mott formula (Eq. (5) in the Methods section) to evaluate the first term on the right-hand side (RHS) of Eq. (4). (2) For a given temperature, the difference between this term and the actual TR, which is a function of the chemical potential, is proportional to $G(\mu ,T)$. We denote this proportionality constant A(T) (note that $A(T)$ is the only fitting number required, for a given temperature, to map the two functions on top of each other). (3) Given the obtained $A(T)$, the difference in entropy between the valleys is then given by $\Delta {S}_{N\to N+1}=2d\left[T\times A(T)\right]/dT$. (A step by step description of the fitting process is detailed in Supplementary Note 3).

In the following we demonstrate the usefulness of this formalism in model systems, where one can compare the entropy obtained using the above relation to that calculated directly from thermodynamic considerations, and finally we apply our formalism to available experimental data.

### Comparison to numerical calculations

**Fig. 1:**
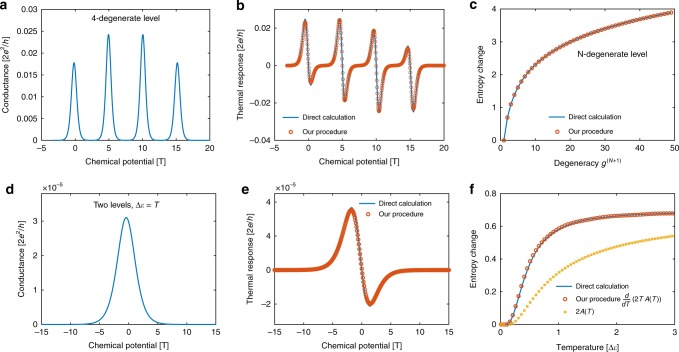
Demonstration of the fitting procedure. **a**, **b** Transport coefficients through a four-fold degenerate quantum dot, calculated via Eq. (1): **a** Conductance, **b** TR (solid blue line) with comparison to the derived expression [Eq. (4)] (red circles). The degeneracies for $n=0,1,2,3,4$-electron many-body states are ${g}^{(N)}=1,4,6,4,1$, respectively (Each peak was separately fitted). **c** Entropy change between two valleys with first valley degeneracy ${g}^{(N)}=1$, as a function of second valley degeneracy ${g}^{(N+1)}$, calculated using the proposed procedure (red circles) compared to the exact result $\mathrm{log}{g}^{(N+1)}$(solid blue line). **d**–**f** Transport through a $U\to \infty$ QD with 2 single-particle non-degenerate interacting levels, separated by $\Delta \epsilon =T$, calculated via Eq. (1): **d** Conductance, **e** TR (solid blue line) with comparison to the derived expression [Eq. (4)] (red circles). **f** Entropy change between the two valleys as a function of temperature. Direct thermodynamic calculation of entropy change (solid blue line) is compared to our procedure ($d2T\times A(T)/dT$) (red circles). $A(T)$ is shown as yellow crosses.

Let us start with a simple example where in each $N$-electron subspace there are ${g}^{(N)}$ degenerate $N$-particle states of energy ${E}^{(N)}$, and all other states can be ignored (i.e., the level spacing is much higher than temperature). In this case the entropy ${S}_{N}$ in each valley is equal to ${\mathrm{log}}\,{g}^{(N)}$, and is temperature independent. Correspondingly, one indeed finds that the proportionality constant is temperature independent, $A(T)={\mathrm{log}}\,({g}^{(N+1)}/{g}^{(N)})/2$. Figure 1b illustrates the correspondence between the TR obtained directly, using Eq. (1), and that obtained by the RHS of Eq. (4) (red circles), for a four-fold degenerate interacting QD, relevant, for example, to a carbon nanotube QD (see also experimental section below). The conductance used in evaluating both terms in the RHS of Eq. (4) was also obtained via Eq. (1) (and is shown in Fig. 1a). In this case there are 4 CB peaks, separating valleys with degeneracies ${g}^{(N)}=1,4,6,4$ and $1$ for $N=0,1,2,3$ and $4$. In order to construct the estimate for the TR in Fig. 1b we have used the above fitting procedure separately for each peak, as the entropy difference between consecutive valleys is different for each peak. The figure displays an almost perfect agreement between the direct calculation of the TR and that obtained by our Ansatz.

In this case, as the entropy change $\Delta S$ between the valleys is temperature independent, the estimate of $A$ at a single temperature is directly proportional to the entropy change through $\Delta S=2A$. In particular, the entropy change across the first CB peak is a direct measure of the degeneracy of the QD ($4$ in the above example). We have repeated the procedure for QDs of arbitrary degeneracy. Figure 1c depicts the entropy change deduced using our procedure (red circles), compared to the expected change in entropy (${\mathrm{log}}\,{g}^{(N+1)}$). We see a perfect agreement even up to large degeneracies. As mentioned above, some aspects of this simple case of a single degenerate level have been addressed before, and it has been suggested that the thermopower through a single-level QD can be used, e.g., to deduce the nature of the neutral modes in the fractional quantum Hall regime^3^.

The advantage of our procedure lies in its application to a multi-level mesoscopic system, such as a multi-level QD, or to a multi-dot system, where the entropy is temperature-dependent. As an example, let us consider the case of two singly degenerate levels, with level spacing $\Delta \epsilon$ (describing, for example, a single-level QD in a magnetic field). One expects that when $T\ll \Delta \epsilon$ the entropy of the single-electron system will be equal to zero, while for higher temperature, larger than $\Delta \epsilon$, it will increase to $\mathrm{log}2$. As the entropy is temperature-dependent, one has to perform the procedure for all $T$ in order to extract $A(T)$, its derivative, and consequently the entropy. For simplicity, we assume that the transition through one of the levels dominates the transport, so Eq. (4), which corresponds to a transition between specific states, will also reflect the full transport coefficient of the system. As we will demonstrate, even though a single transition dominates the transport, the resulting procedure yields the full entropy change in the system.

Figure 1d and e depict, respectively, the calculated conductance and TR, again using Eq. (1), for a specific temperature, $T=\Delta \epsilon$. Figure 1e also shows the TR derived from our procedure—the fitting leads to $A(T=\Delta \epsilon )$ for this temperature. Repeating the same procedure for many temperatures, one is able to produce the whole curve $A(T)$, and then the entropy change, $\Delta S=2d\left[{\mathrm{TA}}(T)\right]/dT$. The resulting estimate for the entropy change is plotted in Fig. 1f along with the thermodynamic calculation of the entropy change: $\Delta {S}_{N\to N+1}=-\partial \left[{F}_{N+1}(T,\mu )-{F}_{N}(T,\mu )\right]/\partial T$ with ${F}_{N}(T,\mu )$ the free energy of the $N$-electron system. Again we observe excellent agreement between the entropy deduced in our procedure and the direct calculation. In Supplementary Note 4 we discuss our procedure for the case when several transitions are relevant to the total transport.

**Fig. 2:**
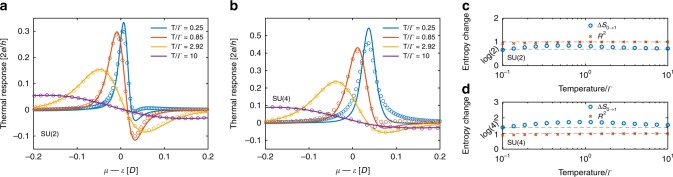
Extension of the procedure to low temperatures. Fitting of the TR obtained directly from NRG (solid line) with TR obtained from Eq. (4) (circles), for the **a** two-fold, and **b** four-fold degenerate quantum dot, in the vicinity of the first CB peak, for various temperatures. **c**, **d** Calculation of the entropy change across the first CB peak for a wide range of temperatures for a **c** two-fold, and **d** four-fold degenerate quantum dot, where the expected entropy changes are $\mathrm{log}2$ and $\mathrm{log}4$, respectively. The closeness of the ${R}^{2}$ estimate of the fitting procedure (crosses) to unity indicates the excellent agreement between the two curves of TR, as shown in **a**, **b**. The $x$-axis in **a**, **b** is in units of $D$, half the bandwidth in the leads, and $\Gamma =0.01D$ and $U=D$ in all three panels.

Interestingly, while this formalism was derived for the weak-coupling ($\Gamma \ll T$) regime, empirically its validity extends outside this strict regime. Since Eq. (1) does not apply to the regime $\Gamma\, \gtrsim\, T$, we have employed here the numerical-renormalization-group (NRG) method (see Methods), which is accurate down to zero temperature. Figure 2 demonstrates the validity of our formalism and shows that the estimates of the entropy, using our procedure for the cases of a two-fold (${\mathrm{SU}}(2)$) and four-fold (${\mathrm{SU}}(4)$) degenerate single-level QD, agree with expected values ($\mathrm{log}2$ and $\mathrm{log}4$, respectively), down to $T\simeq 0.1\Gamma$. The fitting procedure that corresponds to Eq. (4) remains accurate throughout the presented region of temperatures with coefficient of determination (${R}^{2}$) values of close to unity (crosses in Fig. 2c, d). Thus, at least for these two models, our approach extends to couplings to the leads $\Gamma$, which are of the order or even larger than temperature.

### Application to experiments

**Fig. 3:**
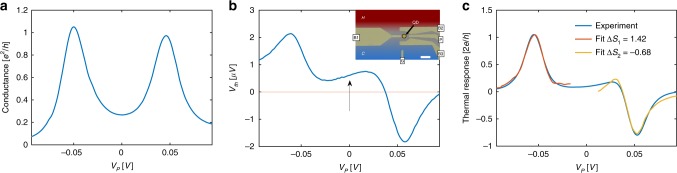
Fitting of the experimental data. **a**, **b** Experimental measurements of **a** conductance and **b** thermovoltage through the QD device, depicted in false color in the inset to (**b**). The horizontal axis corresponds to the QD energy, obtained from multiplying the plunger gate voltage ${V}_{P}$ with gate lever arm $\alpha$ (see methods), and shifting the point of zero energy to the center of the Coulomb-blockade valley. The thermovoltage has a non-zero value in the middle of the valleys around the apparent particle-hole symmetry point (arrow). **c** Fitting procedure [Eq. (4)], performed directly on the experimental data where each peak was fitted separately.

One of the main advantages of our approach, compared, e.g. to that of ref. ^7^, is that it can be readily applied to any previous transport experiment in a mesoscopic system, for which conductance and TR data are available. As an example of the usefulness of the suggested procedure, we have analyzed recent thermoelectric measurement results^21^ through a QD device, formed in a two dimensional electron system of a GaAs/AlGaAs heterostructure using split-gate technology. This technology allows for a high degree of control over system parameters such as QD energy and tunnel coupling $\Gamma$ between the QD and the reservoirs, by adjusting the voltages applied to the split gates. The sample is shown in the inset to Fig. 3b. Gates B1, B2, and B3 are used to form the QD (yellow dot). The tunnel coupling between the QD and the reservoirs H and C can be controlled symmetrically adjusting the gate voltage applied to gate B1. Gate P, the so-called plunger gate, is used to continuously tune the electrochemical potential of the QD, and consequently the number of electrons on the QD. Gate G is not used in these experiments and is kept at ground at all times.

The sample is cooled down in a dilution refrigerator, with an electron base temperature of $\approx\! 230$ mK, in the presence of a small perpendicular magnetic field (B = 0.6 T)^22^. In order to establish a temperature difference $\Delta T$ across the QD, a small heating current was applied to reservoir H (see Methods section and Supplementary Note 5), thereby mainly enhancing the electron temperature in that reservoir. The thermovoltage ${V}_{th}$ is then obtained by recording the voltage drop across the QD as a response to the temperature increase in reservoir H under open circuit conditions (see methods section and Supplementary Note 5 for further details), thus ${V}_{{\mathrm{th}}}={\rm{TR}}\times \Delta T/G$.

**Fig. 4:**
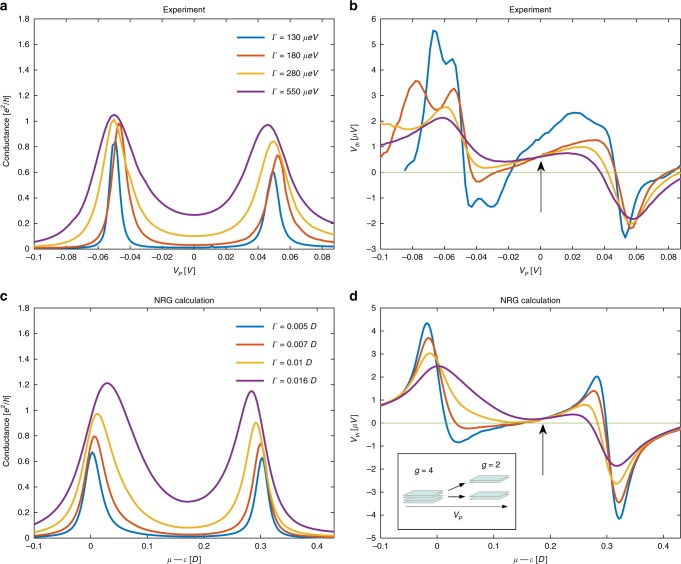
Correspondence between numerical and experimental data for different couplings. Experimental measurements of **a** conductance and **b** thermovoltage through the same device as in Fig. 3, for several values of tunneling widths $\Gamma$. The anomalous nonzero value of the crossing point of the TR curves is denoted by an arrow (due to experimental ambiguity of reference chemical potential, the different curves were aligned so that the apparent particle-hole symmetry point is shifted to ${V}_{P}=0$). Theoretical NRG calculations of **c** conductance and **d** thermopower through a QD with two spin-degenerate levels, with linearly varying level spacing, depicted in the inset to (**d**). The numerical plots were shifted horizontally so that the minima inside the valley for all plots coincided for alignment as in the experimental plots. The results also indicate a non-zero crossing point (arrow). The $x$-axes in **c** and **d** as well as $\Gamma$ are in units of $D$, half the bandwidth in the leads, and we used $U=0.3D$.

Figure 3a, b depict the experimental data for $G$ and ${V}_{{\mathrm{th}}}$, respectively, for a pair of CB peaks. Interestingly, the data show that at points of apparent particle-hole symmetry in the conductance (e.g., arrow in Fig. 3b and crossing point in Fig. 4b), ${V}_{{\mathrm{th}}}$ does not vanish as would be expected from the usual, spin-degenerate QD, described by the standard single-impurity Anderson model^23^. This experimental observation (see also refs. ^16,17^) is to this day an unresolved puzzle in the field (see ref. ^24^ for an attempt to resolve this puzzle).

In the following we detail our analysis of these CB peaks. It has been noted before^17^ that under the condition of heating one reservoir, the actual temperature of the QD can differ greatly from the fridge’s temperature. Since in the present case where $T\,<\,\Gamma \simeq 550\,\mu eV$, the actual temperature cannot be deduced from the width of the CB peaks, we use the temperature as an additional fitting parameter. In addition, since the *x*-axis relation between the conductance measurement (Fig. 3a) and the thermovoltage measurements (Fig. 3b) were not experimentally established, another fitting parameter is introduced: the *x*-axis shift in the measured conductance relative to the measured thermovoltage. The results of fitting the TR to Eq. (4) are depicted in Fig. 3c. As can be seen in the figure, there is a good agreement between the fit and the observed TR in the vicinity of each peak, again using only a few fitting parameters to fit the whole curve (see Supplementary Note 3 for a detailed step-by-step of the analysis of the experimental data using our procedure), illustrating the experimental validity of our approach. Due to the limited availability of the data we used $G(\mu ,T)$ instead of $G(\mu ,{\gamma }_{2}T)$ to estimate ${{\rm{TR}}}^{NI}$. However, this should make a little difference when $T\,<\,\Gamma$.

In applying our method to the experiment, one needs to translate the measured ${V}_{{\mathrm{th}}}$ to the thermoelectric response TR by dividing by $\Delta T$. This value, however, is not easily and accurately determined in an experiment and thus leads to uncertainties in the absolute values of the entropy changes across the peaks. On the other hand, the ratio of these entropy changes across consecutive peaks is independent of $\Delta T$, and is found to be $-2.07\pm 0.13$ for the two peaks depicted in Fig. 3 (the errors estimate is due to variation in possible fitting region around the peaks, see Supplementary Note 3). The simplest scenario giving rise to such a ratio, is that the entropy change across the first peak is $\mathrm{log}4$ while the second is $-\mathrm{log}2$. This means that the first peak signals a transition into a four-fold degenerate state, while the second peak may either correspond to a transition from a four-fold degenerate to a two-fold degenerate state, or from a two-fold degenerate state to a non-degenerate state. This suggests a deviation from the naive picture of consecutive filling of a four-fold degenerate state. Including this scenario into our fit, $\Delta T$ is found to be $\approx\! 20\ mK$, which is close to the experimental estimate of being of the order of 30 mK (see methods and Supplementary Note 5).

While the degeneracy of these two levels seems fortuitous, such a model, in fact, has been claimed to be generic for transport through QDs^25–27^, and has been invoked to explain the repeating phase jumps in the transmission phase through such a dot^28,29^. In these works this is caused by two overlapping levels with different tunneling widths. At each conductance valley the narrow level is filled by an additional electron, shifting the energies of the narrow and the wide level differently, thus leading naturally, due to the degeneracy, to the entropy change of $\mathrm{log}4$ across the first peak. In this scenario, after the second conductance peak the narrow level is doubly occupied, and does not play an additional role in transport, while the wide level is shifted up to overlap with another narrow level, and the process repeats itself. This explained the repeated phase change across consecutive conductance peaks^28,29^, and is, in fact, consistent with the observation that the upshift of the TR from zero at the apparent particle-hole symmetric point happens in consecutive pairs of conductance peaks^16^.

Experimentally, one can easily change the tunneling rates $\Gamma$ between the QD and the leads through the split-gate technique. These data, depicted in Fig. 4a, b, can then be used to differentiate between these possible scenarios. We found that the model that best reproduces the experimental findings, is that of a QD with two spinful states with an energy difference $\Delta \epsilon$ that depends on gate voltage (in the model we used the same $\Gamma$ for both levels to avoid additional parameters). Similar evolution of the degeneracy as a function of chemical potential has already been observed in quantum nano-tubes^30^.

In this model, around the gate voltage corresponding to the first peak (QD energy $\epsilon \sim -0.75$ meV), the two levels are almost degenerate yielding a net four-fold degeneracy(${g}_{N}=0,{g}_{N+1}=4$), which is lifted as the gate voltage is tuned toward the second peak, around QD energy $\epsilon \sim 0.75$ meV (${g}_{N}=2,{g}_{N+1}=1$) (as illustrated in the inset of Fig. 4d). This interpretation leads to the observed values of entropy change.

Figure 4c, d depicts NRG calculation of a specific model for various values of $\Gamma$, where the energy difference between the levels changes linearly with chemical potential, $\Delta \epsilon =a+b(\mu -\epsilon )$, with $a=-0.01D,b=0.13$ ($D$ the bandwidth of the leads). The model reproduces the essential experimental features and those captured by varying $\Gamma$. Some features in the experimental data, such as small side peaks in the lower two values of $\Gamma$, attributed to excited states^31^, are not captured within the current simple model. Interestingly, this model naturally reproduces the non-zero value of the TR at the seemingly particle-hole symmetric point, which is also visible in the experimental data (crossing point in Fig. 4b, marked by an arrow). This anomalous increase of the TR around the middle of the valley is attributed to a non-trivial degeneracy, thus providing a natural explanation that this value of gate voltage does not correspond, in fact, to a particle-hole symmetric point. (An alternative explanation, based on non-linear effects, was suggested in recent work^24^).

## Discussion

In this work, we have derived a theoretical connection between the entropy and transport coefficients in mesoscopic junctions. This connection relates the TR of a Coulomb-blockaded mesoscopic system with arbitrary many-body levels to the conductance and the entropy change between adjacent CB valleys. While the derivation was introduced for weak-coupling $\Gamma$ between the system and the leads (in comparison with temperature), we have demonstrated numerically that, for the case of 2-fold and 4-fold degenerate QD, the method is accurate also for temperatures well below $\Gamma$. This allowed us to apply the method to experimental data in that regime, which yielded non-trivial, and in fact unexpected information about the entropy in each CB valley. The deduced theoretical model, which described the experimental QD, reproduced the measured thermopower and resolved the long-standing puzzle of a finite TR in the apparent particle-hole symmetric point.

The success of this procedure suggests possible venues to extend this analysis especially towards the study of entropy of exotic states. One direction would be to extend the method to low temperatures, thus enabling the determination the degeneracy of the ground state of the full system. This, for example, is particularly relevant to exotic phases, such as the two-channel Kondo system, where the zero temperature entropy is non zero. If the TR of this system can be utilized to deduce the entropy of the ground state, this can be a smoking gun for the observation of the two-channel Kondo ground state^32^ or other such non Fermi liquid ground states. Such an extension has also been suggested in parallel by Sela et al.^33^ to measure the fractional entropy of Majorana zero modes.

## Methods

### High-temperature Mott relation

In relating the non-interacting conductance and TR we use a high-temperature adaptation of the Mott relation^15^.

5 $${{\rm{TR}}}^{NI}(\mu ,T)={\gamma }_{1}T\frac{d{G}^{NI}(\mu ,{\gamma }_{2}T)}{d\mu },$$

where the superscript $NI$ denotes a non-interacting system, and ${\gamma }_{2}=2/\sqrt{3},{\gamma }_{1}=2{\gamma }_{2}^{3}$ are universal values related to properties of the Fermi function (for derivation see Supplementary Note 2).

### Numerical-renormalization group

For the density-matrix numerical-renormalization group (DM-NRG) results we used the open-access Budapest Flexible DM-NRG code^34,35^. The expectation values and the transmission spectral function, required for the evaluation of the conductance through the double dot device^18^, were calculated, assuming, for simplicity, equal couplings to the left and right leads, $\Gamma =\pi \rho {V}^{2}$, and equal and constant density of states $\rho =1/2D$ in the two leads, with a symmetric band of bandwidth $2D$, around the Fermi energy. The NRG simulation is able to output the many-body discrete energy states that the system can occupy and their respective spectral weight, ${\epsilon }_{i},{w}_{i}$. Transport coefficient are then calculated using $G(\mu ,T)=\Gamma \pi \sum {w}_{i}df({\epsilon }_{i}-\mu ,T)/d\mu$ and ${\rm{TR}}(\mu ,T)=\Gamma \pi \sum {w}_{i}df({\epsilon }_{i}-\mu ,T)/dT$.

### Experiment

Our sample is designed similar to the one used by Scheibner et al.^16^. The electron reservoir H, which serves as a hot lead for the quantum dot in our thermopower experiments is shaped into a channel of width $w=2\,\mu m$ and length $l=20\,\mu m$ (see Supplementary Fig. 5). The QD is situated on one side of the channel, delimited by gates B1 and B2,while the opposite side of the channel is delimited by the two gates Q1 and Q2, forming a quantum point contact (QPC), which is positioned exactly opposite to the quantum dot. The QPC is adjusted to the conductance plateau at G = 10 ${e}^{2}/h$. It separates the heating channel H from the reservoir REF, which is kept at ground potential. At the two ends of the heating channel (separated by the distance $l=20\,\mu m$) the 2DES opens up quickly into large reservoirs. The channel can be contacted electrically through two Ohmic contacts ${I}_{1}$ and ${I}_{2}$. We apply a heating current ${I}_{h}=70\, nA$ to the channel, which is modulated at a low frequency $\omega =13$ Hz. Because at low-temperature electron-electron scattering is the dominant scattering mechanism on length scales up to several 10 $\mu$m in our system, the power ${P}_{h}$ introduced through ${I}_{h}$ is dissipated inside the channel only into the electron gas while in the larger reservoirs outside the channel, ${P}_{h}$ is dissipated into the lattice through electron-lattice interaction. From here the heat gets removed efficiently by the dilution refrigerator. In this manner we establish a locally enhanced electronic temperature in the channel while the rest of the 2DES remains approximately at base temperature. Using the thermopower of the QPC as a thermometer^36^ we estimate that for the given ${I}_{h}$, ${T}_{el}$ in the channel increases by $\Delta T\approx 30$ mK. We note that because ${I}_{h}$ gets modulated with $\omega$, the temperature in the heating channel oscillates with $2\omega$ since the dissipated power ${P}_{h}\propto {I}_{h}^{2}\propto {\mathrm{si{n}}}^{2}(\omega t)\propto {\mathrm{cos}}(2\omega t)$. This provides all temperature-driven effects with a clear signature of an oscillation frequency of $2\omega$. The thermovoltage ${V}_{th}$ of the QD is obtained by measuring the potential difference between the contacts of the two cold reservoirs ${V}_{\mathrm{{ref}}}$ and ${V}_{C}$ using a Lock-In amplifier operating at $2\omega =26\ Hz$. Since the QPC is adjusted to a conductance plateau, its contribution to the ${V}_{th}$ is zero. Hence the measured signal can be attributed fully to the QD. In order to suppress any potential fluctuations at $\omega$ in close vicinity to the QD structure, which may occur due to unwanted capacitive coupling inside the sample, we let the excitation voltage for the heating current at both contacts of the heating channel oscillate symmetrically with respect to ground. Since reservoir REF is kept grounded, this suppresses oscillations of the electrical potential at $\omega$ around the QD structure.

## Supplementary information

Supplementary Information

## Data Availability

The datasets generated and analysed in the study are available upon request from the corresponding authors.

## References

[1] Andrei N, Destri C (1984). Solution of the multichannel Kondo problem. Phys. Rev. Lett..

[2] Ben-Shach G, Laumann CR, Neder I, Yacoby A, Halperin BI (2013). Detecting Non-Abelian anyons by charging spectroscopy. Phys. Rev. Lett..

[3] Viola G, Das S, Grosfeld E, Stern A (2012). Thermoelectric probe for neutral edge modes in the fractional quantum hall regime. Phys. Rev. Lett..

[4] Smirnov S (2015). Majorana tunneling entropy. Phys. Rev. B.

[5] Cockins L., Miyahara Y., Bennett S. D., Clerk A. A., Studenikin S., Poole P., Sachrajda A., Grutter P. (2010). Energy levels of few-electron quantum dots imaged and characterized by atomic force microscopy. Proceedings of the National Academy of Sciences.

[6] Hofmann A (2016). Measuring the degeneracy of discrete energy levels using a GaAs/AlGaAs quantum dot. Phys. Rev. Lett..

[7] Hartman N (2018). Direct entropy measurement in a mesoscopic quantum system. Nat. Phys..

[8] Chaikin PM, Beni G (1976). Thermopower in the correlated hopping regime. Phys. Rev. B.

[9] Behnia K, Jaccard D, Flouquet J (2004). On the thermoelectricity of correlated electrons in the zero-temperature limit. J. Phys. Condens. Matter.

[10] Zlatić V, Monnier R, Freericks JK, Becker KW (2007). Relationship between the thermopower and entropy of strongly correlated electron systems. Phys. Rev. B.

[11] Mravlje J, Georges A (2016). Thermopower and entropy: lessons from Sr2RuO4. Phys. Rev. Lett..

[12] Heikes, R. R. & Ure, R. W. *Thermoelectricity: science and engineering* (Interscience Publishers, New York, London, 1961).

[13] Doumerc JP (1994). Thermoelectric power for carriers in localized states: a generalization of Heikes and Chaikin-Beni formulae. J. Solid State Chem..

[14] Peterson MR, Shastry BS (2010). Kelvin formula for thermopower. Phys. Rev. B.

[15] Cutler M, Mott NF (1969). Observation of anderson localization in an electron gas. Phys. Rev..

[16] Scheibner R, Buhmann H, Reuter D, Kiselev MN, Molenkamp LW (2005). Thermopower of a kondo spin-correlated quantum dot. Phys. Rev. Lett..

[17] Svilans A (2018). Thermoelectric characterization of the Kondo resonance in nanowire quantum dots. Phys. Rev. Lett..

[18] Meir Y, Wingreen NS (1992). Landauer formula for the current through an interacting electron region. Phys. Rev. Letters.

[19] Beenakker CWJ (1991). Theory of Coulomb-blockade oscillations in the conductance of a quantum dot. Phys. Rev. B.

[20] Beckel A (2014). Asymmetry of charge relaxation times in quantum dots: the influence of degeneracy. Europhys. Lett..

[21] Thierschmann, H. Thierschmann, H. *Heat Conversion in Quantum Dot Systems*. Thesis (2014).

[22] Van der Wiel WG (2000). The Kondo effect in the unitary limit. Science.

[23] Costi TA, Zlatić V (2010). Thermoelectric transport through strongly correlated quantum dots. Phys. Rev. B.

[24] Karki DB, Kiselev MN (2017). Thermoelectric transport through a SU(N) Kondo impurity. Phys. Rev. B.

[25] Silvestrov PG, Imry Y (2000). Towards an explanation of the mesoscopic double-slit experiment: a new model for charging of a quantum dot. Phys. Rev. Lett..

[26] Silvestrov PG, Imry Y (2001). Spin effects and transport in quantum dots with overlapping resonances. Phys. Rev. B.

[27] Golosov DI, Gefen Y (2006). Transmission through quantum dots: focus on phase lapses. Phys. Rev. B.

[28] Yacoby A, Heiblum M, Mahalu D, Shtrikman H (1995). Coherence and phase sensitive measurements in a quantum dot. Phys. Rev. Lett..

[29] Yacoby A, Schuster R, Heiblum M (1996). Phase rigidity and h/2e oscillations in a single-ring Aharonov-Bohm experiment. Phys. Rev. B.

[30] Pecker S (2013). Observation and spectroscopy of a two-electron Wigner molecule in an ultraclean carbon nanotube. Nat. Phys..

[31] Beenakker CW, Staring AA (1992). Theory of the thermopower of a quantum dot. Phys. Rev. B.

[32] Potok RM, Rau IG, Shtrikman H, Oreg Y, Goldhaber-Gordon D (2007). Observation of the two-channel Kondo effect. Nature.

[33] Sela E (2019). Detecting the universal fractional entropy of majorana zero modes. Phys. Rev. Lett..

[34] Tóth AI, Moca CP, Legeza Á, Zaránd G (2008). Density matrix numerical renormalization group for non-Abelian symmetries. Phys. Rev. B.

[35] Legeza, O., Moca, C. P., Toth, A. I., Weymann, I. & Zarand, G. Manual for the Flexible DM-NRG code. *Preprint at* https://arxiv.org/abs/0809.3143 (2008).

[36] Molenkamp LW, Van Houten H, Beenakker CWJ, Eppenga R, Foxon CT (1990). Quantum oscillations in the transverse voltage of a channel in the nonlinear transport regime. Phys. Rev. Lett..

