# Supplementary material for: How to measure the entropy of a mesoscopic system via thermoelectric transport
Source: Nat Commun. 2019 Dec 20;10:5801. doi: 10.1038/s41467-019-13630-3 (PMC6925120; doi:10.1038/s41467-019-13630-3)
Supplement: Supplementary file 1 — Supplementary Information [file 41467_2019_13630_MOESM1_ESM.pdf]

Supplementary Information

Supplementary Note 1: Derivation of Eqs. 2 and 3

Eqs. 2 and 3 of the main text express the conductance and T of the full interacting system with arbitrary spectrum and wavefunctions, in terms of these quantities for the non-interacting system, an expression which is valid in weak-coupling($\Gamma \ll T$), Coloumb-blockade ($U \gg T$) regime. Following Eq. 1 of the main text, we can generally write the expressions for conductance and TR

$$G(\mu,T) = \sum_{ij} G_{ij} = \sum_{ij} \frac{e^2}{h} \mathscr{T}_{ij}^{(0)} \times (P_i^{(N)} + P_j^{(N+1)}) \frac{df(E_j^{(N+1)} - E_i^{(N)} - \mu, T)}{d\mu} \tag{1}$$

$$\mathrm{TR}(\mu,T) = \sum_{ij} \mathrm{TR}_{ij} = \sum_{ij} \frac{e}{h} \mathscr{T} ij^{(0)} \times (P_i^{(N)} + P_j^{(N+1)}) \frac{df(E_j^{(N+1)} - E_i^{(N)} - \mu, T)}{dT}$$

where $\mathscr{T}_{ij}^{(0)} = \sum_{n,m} \Gamma_{ij} \langle \psi_j | d_n^\dagger | \psi_i \rangle \langle \psi_i | d_m | \psi_j \rangle$ and the sum is over all states i and j in the N and $N+1$-electron subspaces, respectively. In the above, we used the fact that for large U the transport properties only involve, at the most, two $N-$electron subspaces, say N and $N+1$. In equilibrium, the probability that the system is in a specific N or $N+1$ many-body state is

$$P_i^{(N)}(\mu,T) = \frac{1}{Z(\mu,T)} \exp(-\frac{E_i^{(N)} - N\mu}{T}), \quad P_j^{(N+1)}(\mu,T) = \frac{1}{Z(\mu,T)} \exp(-\frac{E_j^{(N+1)} - (N+1)\mu}{T}) \tag{2}$$

where $Z = \sum_i \exp\left[(E_i^{(N)} - N\mu)/T\right] + \sum_j \exp\left[(E_j^{(N+1)} - (N+1)\mu)/T\right]$ is the partition function.
We define the function $K_{ij}(\varepsilon - \mu, T)$ by the relation

$$\frac{d}{d\mu} K_{ij}(\varepsilon - \mu, T) = (P_i^{(N)} + P_j^{(N+1)}) \frac{df(\varepsilon - \mu, T)}{d\mu}. \tag{3}$$

The main observation is that, since the factor $(P_i^{(N)} + P_j^{(N+1)})$ approaches a constant in the limits $\mu \to \pm\infty$, and $f(\varepsilon - \mu, T)$ drops, as a function of μ, from unity to zero on a scale of T, the function K will also change from a constant to zero on the scale of T, and thus can be well described by a, possibly shifted, Fermi function, $K_{ij}(\varepsilon - \mu, T) \simeq C(T) f(\varepsilon - \mu - \Delta_{ij}(T), T)$. This Ansatz allows one to write, for the relevant conductance term

$$G_{ij}(\mu,T) \simeq C(T) \mathscr{T}_{ij}^{(0)} \frac{d}{d\mu} f(E_j^{(N+1)} - E_i^{(N)} - \mu - \Delta_{ij}(T), T). \tag{4}$$

Supplementary Eq. 4 is identical, up to a multiplicative constant, to the conductance of a non-interacting system with the same spectrum and matrix elements, but a shifted chemical potential (Eq. 2 of the main text). In order to find $\Delta_{ij}(T)$ one has to compare the positions of the peaks of $dK/d\mu$ and $df/d\mu$. Before deriving it for the general case, let us look at a simple case – an M-level QD, in which all the M single-electron levels are degenerate with energy ε and repel one another by the Coulomb repulsion U. In this system the energy of the N-electron many-body state is $E^{(N)} = N\varepsilon + N(N-1)U/2$, and he degeneracy of each many-body energy is $g^{(N)} = \binom{M}{N}$ (the i, j indices have been omitted, since all the degenerate states were assumed to have the same coupling). The partition function is given by $Z = g^{(N)} \exp\left[(E^{(N)} - N\mu)/T\right] + g^{(N+1)} \exp\left[(E^{(N+1)} - (N+1)\mu)/T\right]$.
The equation for the function K can be solved analytically to give $K(\varepsilon, T) = \log\Big(1 + \big[h-1\big] f(\varepsilon, T)\Big)/\log(h)$, with $h = (g^{(N+1)}/g^{(N)})$. This function can be well approximated by $f(x - \Delta(T), T)$, with $\Delta(T) = T\log(h)/2$. Supplementary Fig. 1a depicts the first Coulomb blockade peak in the conductance through such a QD, with varying degeneracies, using the full expression (Supplementary Eq. 1) and the Ansatz (Supplementary Eq. 4) with excellent agreement, though there are noticeable deviations for degeneracy ratios $h \gtrsim 5$, which are usually physically irrelevant.
Following the same procedure for the general case, one can again calculate Δ_{ij} analytically, which results in Eq. 3 of the main text.
While the expression for the conductance looks exactly like that of a non-interacting system with transmission coefficient $\mathscr{T}_{ij}^{(0)}$ and a shifted chemical potential, $\mu \to \mu + \Delta_{ij}(T)$, the temperature dependence of the shift Δ will lead to an additional contribution to the TR from $d\Delta_{ij}(T)/dT$ (Eq. 4 of the main text). It is this additional contribution that allows us to determine

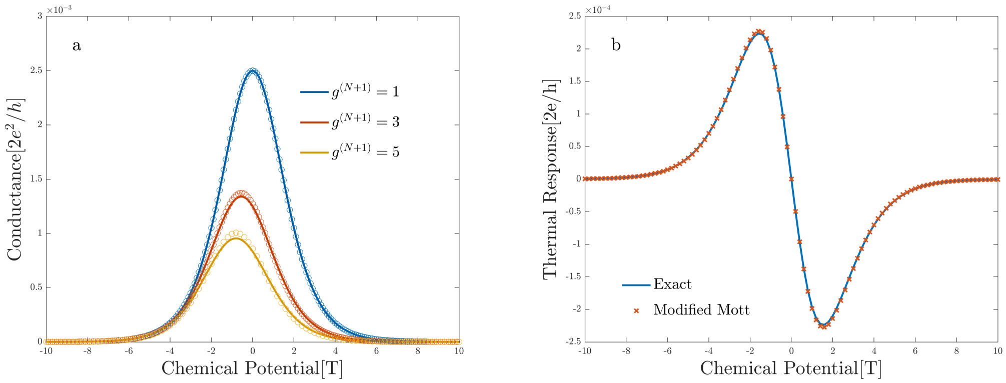

Supplementary Figure 1. Validity of Supplementary Eq. 4 and the high temperature Mott relation (Supplementary Eq. 7). (a) Conductance through a quantum dot as a function of chemical potential in the vicinity of the $N \to N+1$ peak for various degeneracies $g^{(N+1)}$ with $g^{(N)}$ kept at 1. The solid line is the exact calculation (Supplementary Eq. 1) while the circles are a calculation with an effective Fermi function (Supplementary Eq. 4). The prefactor C(T) for every curve was found by fitting. (b) The high-temperature Mott relation illustration: direct calculation of TR (solid line) and calculation of TR using the modified Mott relation (crosses) as a function of chemical potential (in units of temperature) in a system with a single level with energy $\varepsilon = 0$.

the entropy difference (see main text and below). Explicitly, the TR can be treated similarly to the conductance (Supplementary Eq. 4), where the derivative with respect to μ is replaced by derivation with respect to temperature. This can be rewritten as

$$\mathrm{TR}_{ij}(\mu,T) \simeq C(T)\mathscr{T}_{ij}^{(0)}\frac{\partial}{\partial T}f(E_j^{(N+1)}-E_i^{(N)}-\mu-\Delta_{ij}(T),T)+\frac{d\Delta_{ij}(T)}{dT}C(T)\mathscr{T}_{ij}^{(0)}\frac{d}{d\mu}f(E_j^{(N+1)}-E_i^{(N)}-\mu-\Delta_{ij}(T),T). \quad (5)$$

where the partial derivative in the first term indicates that the shift in the chemical potential is treated as a constant in T. The first term can be identified as a non-interacting TR shifted along the chemical potential and the second term can be identified as the non-interacting conductance times $d\Delta_{ij}(T)/dT$, which results in Eq. 4 in the main text.

Supplementary Note 2: Derivation of the high-temperature Mott formula

The standard Mott relation[1] relates the derivative of the conductance to the TR at low temperatures. This relation relies on an approximate relation, valid at low temperatures, between $df(\varepsilon-\mu,T)/dT$, which appears in the expression for the TR, to $d^2f(\varepsilon-\mu,T)/d\mu^2$, which, for the non-interacting system, is related to $dG/d\mu$. The relation between these two functions relies on the Sommerfeld expansion, which assumes that the temperature is the smallest energy scale in the problem. In the present case, where $T \gg \Gamma$, this relation has to be modified. In the following we derive an alternative relation between these two functions, valid in this regime, which will then be used to obtain an estimate of the TR for the non-interacting system. As mentioned above, and in the main text, the deviation of the true TR from the non-interacting TR, estimated by the high-temperature Mott formula, allows us to determine $d\Delta/dT$, and as a consequence, the entropy.
One can expand the Fermi function as follows

$$\begin{aligned} d^2f(\varepsilon-\mu,T)/d\mu^2 &= \frac{x}{8T^2}-\frac{x^3}{24T^2}+O(x^5) \\ df(\varepsilon-\mu,T)/dT &= \frac{x}{4T}-\frac{x^3}{16T}+O(x^5) \end{aligned} \quad (6)$$

where $x=(\varepsilon-\mu)/T$. These two functions can be made equal up to third order in $(\varepsilon-\mu)/T$ by determining two parameters in the relation between these two functions such that $df(\varepsilon-\mu,T)/dT = T\gamma_1 d^2f(\varepsilon-\mu,\gamma_2 T)/d\mu^2$. These factors are an overall factor $\gamma_1 = 2\gamma_2^3$ and a temperature factor $\gamma_2 = \frac{2}{\sqrt{3}}$. Both these factors do not depend on the system in question, as they only depend on the properties of the Fermi function. As a result of this relation, the high-temperature Mott relation, relying on the fact that transport coefficients are solely governed by their respective Fermi functions and valid only for non-interacting systems, is given by:

$$\mathrm{TR}^{NI}(\mu,T) = \gamma_1 T\frac{dG^{NI}(\mu,\gamma_2 T)}{d\mu}, \quad (7)$$

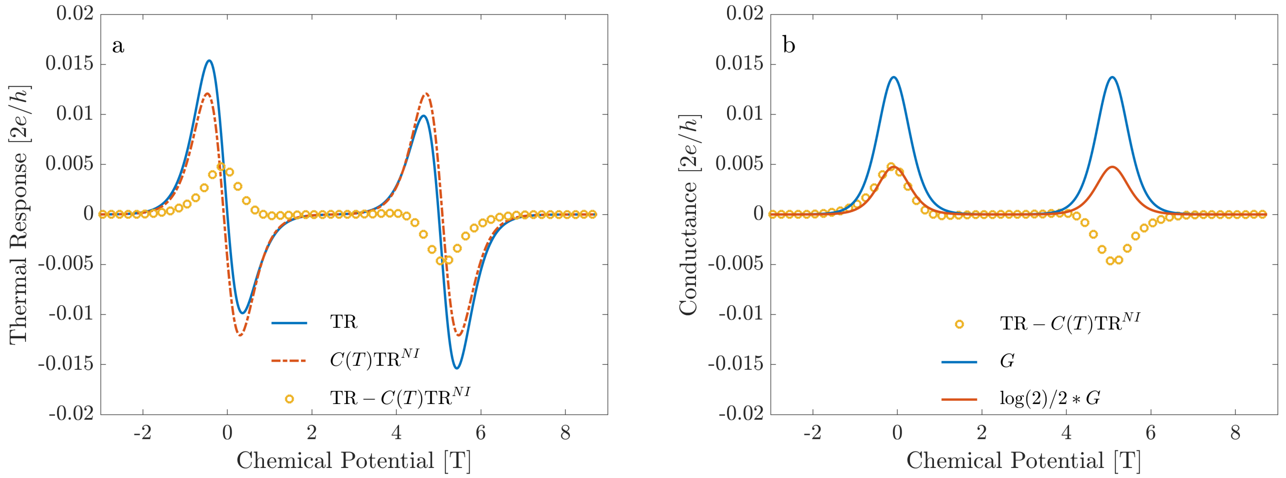

Supplementary Figure 2. The different components of the thermal response (Supplementary Eq. 5). (a) the full TR (solid blue line), Mott contribution (dot-dash red line) and their difference (yellow circles), illustrating the various terms in Supplementary Eq. 8. (b) Comparison of the difference between the full TR and the Mott contribution, and the scaled conductance, as per the second term of the RHS of Supplementary Eq. 8

which is quoted as Eq. 5 in the main text. Supplementary Fig. 1b illustrates the excellent agreement between the real TR, calculated using Supplementary Eq. 1, and the TR derived from the calculated conductance through the high temperature Mott relation adaptation, for a noninteracting system. This relation allows us to estimate the first term on the RHS of Eq. 4 of the main text from measurable quantities, such as the conductance. Note, however, that the relation (7) relates $\mathrm{TR}^{NI}(T)$ to the conductance at temperature $\gamma_2 T$.

Supplementary Note 3: Fitting to numerical and experimental data

The Mott type contribution (Eq. 5 and Supplementary Eq. 7) relates the TR at temperature T to the derivative of the conductance G at temperature $\gamma_2 T$. Since the actual G and TR, at temperature T and chemical potential μ, are related to the non-interacting G and TR at temperature T and chemical potential $\mu+\Delta(T)$, this leads to a small relative chemical potential shift $\Delta(\gamma_2 T)-\Delta(T)$ between these quantities. Since $\Delta(T)$ can be deduced from the fitting parameter $A(T)$, this shift can be included self-consistently in the fitting procedure. In addition, the temperature dependent prefactor $C(T)$ in Eq. 2 of the main text, introduces a small factor between the two transport coefficients: $C(\gamma_2 T)/C(T)$. We found that this factor was very close to unity and we did not fit it in our procedure, since it only improves the result negligibly.
Explicitly, the fitting procedure delineated in the main text can be summarized with the fitting formula:

$$\mathrm{TR}(\mu,T)=\gamma_1\frac{\partial}{\partial T}G(\mu-\left[\gamma_2 TA(\gamma_2 T)-TA(T)\right],\gamma_2 T)+A(T)G(\mu,T) \tag{8}$$

where the only fitting parameter, for every single value of temperature, is $A(T)$.
We carried out this procedure separately in the vicinity of each peak , as the value of A is different for each peak. The "vicinity of each peak" is defined between the points where the TR and conductance vanish. In order to illustrate the various components of Supplementary Eq. 8 (and Eq. 4 in the main text), we plot in Supplementary Fig. 2a the full TR (solid blue line) for the case of a doubly degenerate level with $U\to\infty$, along with the fitting of the first term on the RHS of Supplementary Eq. 8, the high temperature Mott contribution (the dot-dash red line). The difference between what would be expected in a non-interacting system (the Mott contribution) and full TR (shown in yellow circles) corresponds to the second term on the RHS of Supplementary Eq. 8, which is evidently just a numerical factor times the conductance, as seen in Supplementary Fig. 2b. That numerical factor is A, which in this case is half the entropy change across each peak.
In the case of a temperature dependence in $A(T)$, such as that depicted in Figs. 1e,f, the self consistent procedure involves an iterative procedure. We infer $A(T)$ neglecting the contribution of $dA(T)/dT$ to the shift, and then use that inferred value to estimate the derivative needed to calculate the correct shift in chemical potential. In theory, one may continue to iterate, however we find that due to the small effect of this derivative, one iteration is enough, as seen in Figs. 1e,f.
The experimental data and its limitations has offered further challenges. Since experimentally, the two measurements: conductance and thermovoltage, involve different sets of gate voltages, there is an arbitrary shift of the x-axis, and we have added this value as a fitting parameter. In addition, the temperature that was experimentally established without current heating

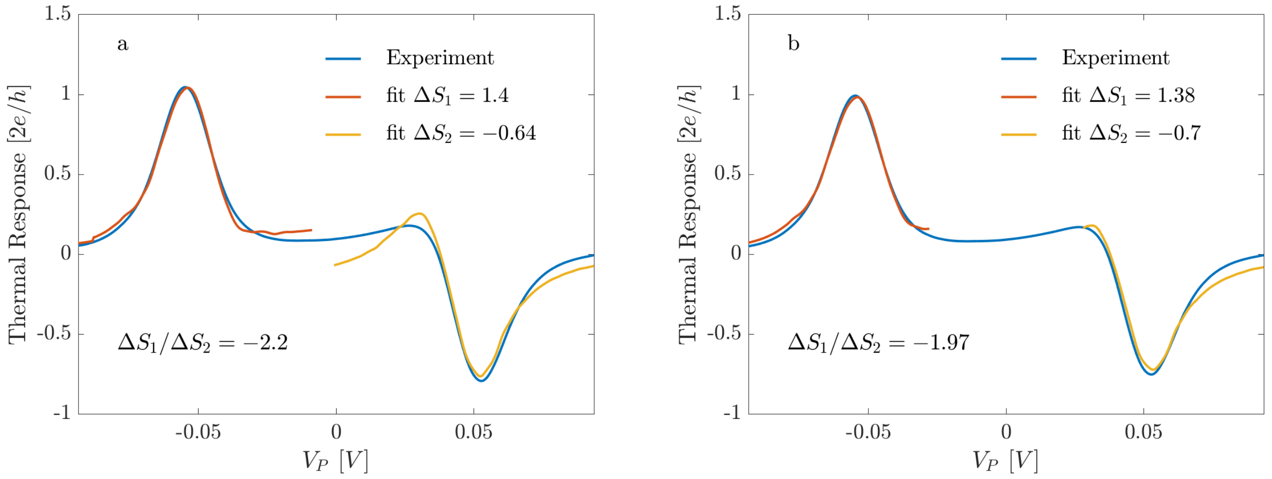

Supplementary Figure 3. Estimation of variance in fitting procedure. Fitting results for experimental TR similar to Fig. 3c in the main text with two extreme choices for fitting regions around the peaks: (a) Fitting the full range to mid point between the peaks and (b) fitting only the bulk of the peak.

is not accurate for the case of an existing temperature bias, so we also added that as a fitting parameter. The resulting fitting equation reads

$$\mathrm{TR}(\mu) = \gamma_1 \frac{\partial}{\partial T} G(\mu - \beta - (\gamma_2 - 1)TA) + G(\mu - \beta)A \tag{9}$$

where A, β, T are the fitted parameters.
The peaks in the experimental data are not clearly separated. Using different definitions of ”peak vicinity” we get slightly different results. In Supplementary Fig. 3 we depict two extreme choices for peak definition, in (a) we show the fit for the whole peak, up to the mid point between the peaks, and in (b) where only the bulk of the peak is fitted. Such fits allow us to determine the extreme values of ratio of entropy changes, which is independent of the value of ΔT, $\Delta S_1/\Delta S_2 = -2.07 \pm 0.12$. Assuming that this corresponds to the likely scenario of $\Delta S_1 = \log 4$ and $/\Delta S_2 = -\log 2$ allows us to deduce $\Delta T = 20.5mK \pm 0.5mK$.

Supplementary Note 4: The case with several conducting levels

Eq. 3 of the main text expresses the shift in the chemical potential for a given transition from state i to state j, Δ_{ij}, as a sum of two terms, the first proportional to the energy difference between the two states, while the second, one half of the total free energy, is independent of the particular states i and j. This shift is obtained, either numerically or experimentally, by dividing the difference between the full TR and that of the corresponding non-interacting system, deduced via the Mott relation, by the conductance. Sec. 3 of the main text considered cases where a transition through single level dominated the conductance. In order to demonstrate the case of mutli-level transport, let us consider the model discussed in Sec. 3, a two-level QD, of non-degenerate levels, where each of the transitions between an empty QD to one of the levels being occupied has a finite contribution to conductance, $G_{11}(\mu,T)$ and $G_{12}(\mu,T)$, respectively. In that case, the procedure will approximately yield $\Delta(T) = \left[G_{11}(\mu,T)\Delta_1(T) + G_{12}(\mu,T)\Delta_2(T)\right] / \left[G_{11}(\mu,T) + G_{12}(\mu,T)\right]$, where $\Delta_i(T)$ is the chemical-potential shift corresponding to the transition through level i (note that $\Delta_2 - \Delta_1 = \Delta\varepsilon/2$. Taking the derivative with respect to T, we find, in addition to the entropy change term ΔS derived before, an additional term

$$\frac{d\Delta(T)}{dT} = \Delta S + \frac{\Delta\varepsilon}{T^2}\left(G_{12}(\mu,T)\frac{dG_{11}(\mu,T)}{dT} - G_{11}(\mu,T)\frac{dG_{12}(\mu,T)}{dT}\right)|_{\mu=\mu_{max}} \tag{10}$$

The second term vanishes when one of the levels dominates the transport, or when the levels are degenerate, or at high temperatures ($T \gg \Delta\varepsilon$) or at low temperatures ($T \ll \Delta\varepsilon$). So the maximal deviation is expected at $T \simeq \Delta\varepsilon$, when the levels have the same coupling to the leads. In this case the deviation is approximately $(\Delta\varepsilon/\Delta T)^2/(1+\cosh(\Delta\varepsilon/T))$.
Supplementary Fig. 4a depicts the deduced $\Delta(T)$ for the two-level case, with different ratios of couplings to the leads. As expected, for intermediate values of the ratio of the couplings, one finds that the high-T value of ΔS is a weighted average of the ΔS_{11} and S_{12}, while at low temperatures, they all converge to S_{11}. Clearly, in the regime $T \simeq \Delta\varepsilon$ the derivative becomes larger. This is manifested in Supplementary Fig. 4b, where the deduced values of the entropy change are depicted, where the

exact result for entropy change is captured for the two cases with only one contributing transition ($\Gamma_{12} = 0$ or $\Gamma_{11} = 0$). When both level contributes, some deviations from the exact results are noticeable. Note, however, that, as the couplings to the leads depend exponentially on energy, it is unlikely that they will be equal to each other, and thus this regime may not be physically relevant.

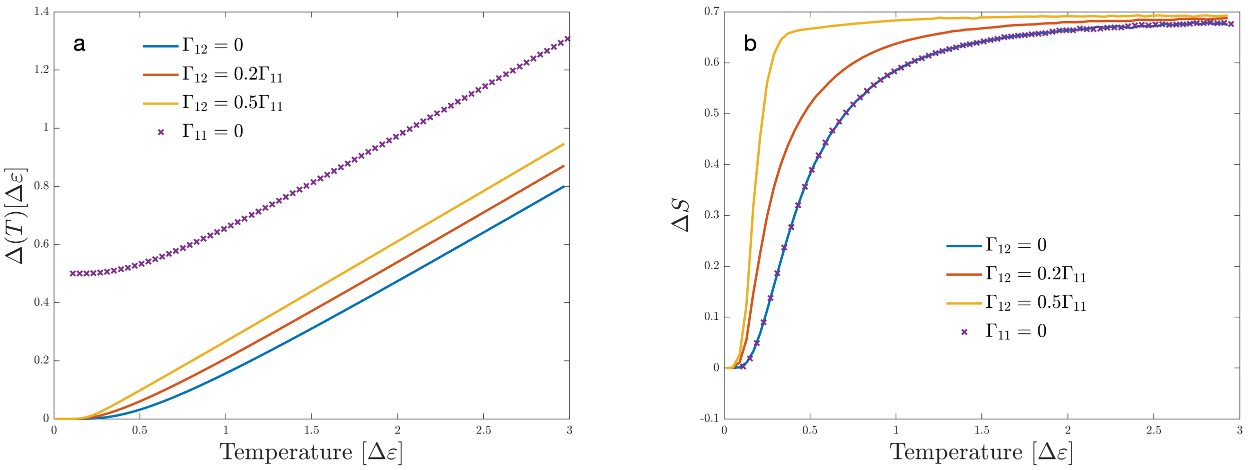

Supplementary Figure 4. Multi-level fitting results. Fitting results for (a) $\Delta(T)$ and (b) the deduced entropy change ΔS for various ratio of coupling Γ_{ij} to the leads. The correct result for deduced entropy is recovered in the two cases of single dominating transition.

Supplementary Note 5: Experimental setup and procedure

The device used in the experiments was fabricated from a GaAs/AlGaAs heterostructure hosting a two dimensional electron system (2DES) 90 nm under the surface. The 2DES has a nominal carrier density $n = -2.98 \times 10^{11} cm^{-2}$ and a mobility $\mu = 550 \times 10^5 cm^2 (Vs)^{-1}$ at T=4 K. The gate pattern was fabricated with standard optical and e-beam lithography and lift-off techniques. The gate electrodes consist of a 2 nm thick Ti adhesion layer and a 50 nm thick Au layer. Ohmic contacts with the 2DES were obtained through annealed Au/Ge pads and bonding.

Supplementary Fig. 5a depicts a drawing of the gate pattern of the sample. The scale bar corresponds to 1 μm. Gates are shown in yellow. Reservoirs which are at low (T_c) and higher ($T_h = T_c + \Delta T$) temperatures during thermopower measurements are denoted with blue and red color, respectively. The green dashed square indicates the region of which an SEM image is shown in the main text. The quantum dot is formed with gates B1, B2, B3, and P. The heating channel H (red) (width $w = 20\mu m$ and a length of $20\mu m$) serves as one of the leads of the QD. It is equipped with two Ohmic contacts I_1 and I_2 through which a heating current I_h is applied to the channel. The other lead of the QD, labeled C, is equipped with a voltage probe V_C. It serves as a cold equilibrium reservoir (blue). The quantum point contact (QPC) formed by the gates Q1 and Q2 is placed exactly opposite to the QD. The QPC is adjusted to the 10 e^2/h conductance plateau. It separates the heating channel from the cold electron reservoir denoted REF. The thermovoltage V_{th} measured between V_{ref} and V_C as a response to a temperature increase in H is then given by $V_{th} = V_C – V_{ref} = (S_{QD} – S_{QPC})\Delta T$. Since the QPC is adjusted to a conductance plateau, its thermopower $S_{QPC} = 0$. Therefore the measured voltage V_{th} can be assigned entirely to the thermopower of the QD, S_{QD}.

For a heating current $I_h = 70$ nA the electron gas in the channel typically heats up by approximately $\Delta T \approx 50mK$ (cf. supplementary section C in Refs.[2,3]). If we take into account that the electron density in our material is higher by approximately a factor 1.4 compared with Ref.[2] ($2.98 \times 10^{11} cm^{-2}$ here and $2.14 \times 10^{11} cm^{-2}$ in Reference[2]) and that the heat capacity of a 2DES follows the carrier density linearly in first approximation[4], we estimate that for $I_h = 70nA$, $\Delta T \approx 30mK$ in our sample.

Supplementary Fig. 5b shows the stability diagram obtained from measurements of the differential conductance dI/dV of the QD with all reservoirs at low temperature. The Coulomb diamonds, signatures of a fixed charge occupation number N of the QD, are highlighted with dashed blue lines. From the size of the Coulomb diamonds on the V_{sd} axis we can extract the charging energy U of the QD, $U \approx 1.7meV$. This allows us to calculate the electrostatic lever arm of the plunger gate voltage $\alpha = 0.016e$ which can then be used to convert the plunger gate voltage axis V_P into QD energies ε using $\varepsilon = \alpha \times V_P$. From Fig. S3b we see that in the Coulomb blockade regions for (N-1) and (N+1) the conductance for small bias voltage is not fully suppressed but it shows a zero bias conductance. This is a signature of Kondo correlations being present for these charge configuration.

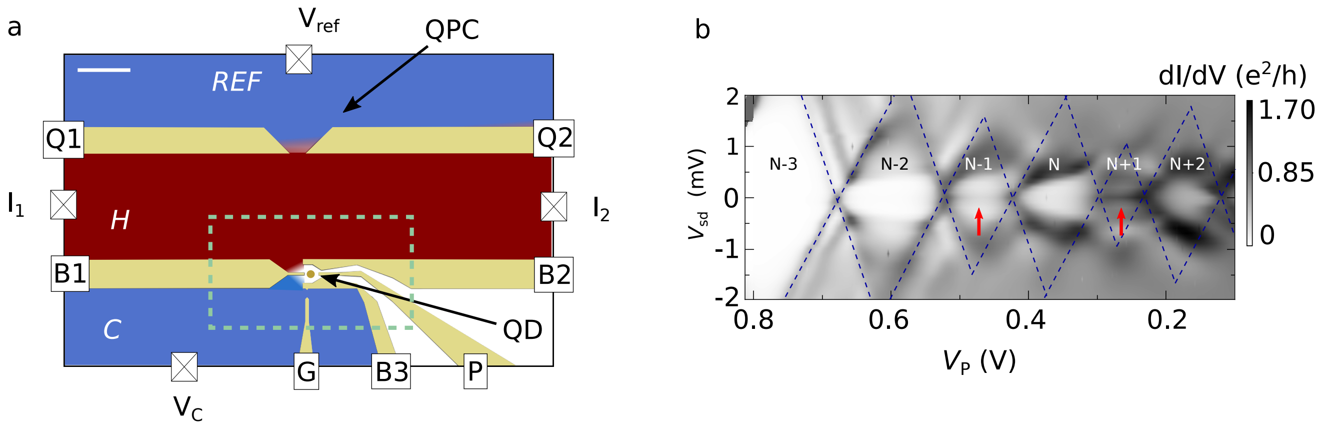

Supplementary Figure 5. Experimental setup and analysis. (a) Gate layout of the sample. The gates (yellow) B1, B2, B3 and P are used to confine the QD (yellow dot), which is tunnel coupled to the equilibrium electron reservoirs H (red) and C (blue). Reservoir H is shaped through the pairs of gates B1, B2 and Q1, Q2 into a channel. Gates Q1 and Q2 further form a quantum point contact used for thermometry. For thermopower measurements a heating current I_h is applied to the heating channel through contacts I_1 and I_2. The thermovoltage of the QD is measured using the voltage probes V_{ref} and V_C, which are both connected to cold reservoirs. The scale bar corresponds to 1 μm. The dashed frame denotes the region of which a SEM image is shown in the main text. (b) Differential conductance dI/dV stability diagram of the QD with all reservoirs at low temperature. The Coulomb diamonds are indicated with dashed lines. The charge occupation number associated with the respective diamonds are denoted (N-3), (N-2), . . . etc. Red arrows denote regions with Kondo enhanced zero bias conductance, indicative for an odd electron occupation number.

Supplementary Note 6: Role of magnetic field

The experimental conductance and thermovoltage data discussed in the main text were measured with a perpendicular magnetic field B = 0.6 T applied. In Supplementary Fig. 6 we show the evolution of the conductance G with magnetic field as a function of plunger gate voltage V_P. We see that for B = 0 (yellow line), the conductance G is close to zero for higher occupation numbers ($V_P > 0.1V$). For smaller occupation numbers ($V_P < 0.1V$), corresponding to the part discussed in the main text, two conductance peaks are visible. As B is increased, transmission through the QD changes dramatically. For $V_P > 0.1V$, two conductance peaks emerge, with a partly suppressed Coulomb blockade valley in between. For $V_P < 0.1V$ the effect of B on QD transmission is less drastic, yet clearly visible from changes in peak shape and height. Similar behavior has been observed for the B dependent transmission of a QD, for example, by van der Wiel et al. [21]. At B = 0.6 T (blue line), G has evolved into a clear, well-defined series of conductance peaks. In order to base our thermopower analysis on solid footing, and remove contributions from QD states with unusually suppressed transmission, we have therefore chosen B = 0.6T as experimental condition to study the thermopower of the system.

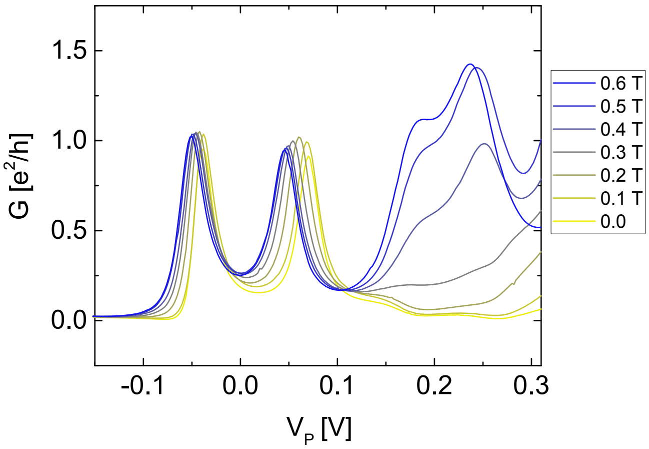

Supplementary Figure 6. Magnetic field dependence of the data. Conductance G as a function of plunger gate V_P for different perpendicular magnetic fields B = 0 to 0.6T. $V_P = 0$ has been set to the center of the conductance valley between the first two conductance peaks for B=0.6T.

Supplementary References

1. Cutler, M. & Mott, N. F. Observation of anderson localization in an electron gas. *Physical Review* **181**, 1336–1340 (1969). arXiv:1011.1669v3.
2. Thierschmann, H. *et al.* Three-terminal energy harvester with coupled quantum dots. *Nature Nanotechnology* **10**, 854–858 (2015). 1603.08570.
3. Thierschmann, H. Heat Conversion in Quantum Dot Systems. *Thesis* (2014).
4. Molenkamp, L. W., Van Houten, H., Beenakker, C. W. J., Eppenga, R. & Foxon, C. T. Quantum oscillations in the transverse voltage of a channel in the nonlinear transport regime. *Physical Review Letters* **65**, 1052–1055 (1990).
